# Modulation of Actin Filament Dynamics by Inward Rectifying of Potassium Channel Kir2.1

**DOI:** 10.3390/ijms21207479

**Published:** 2020-10-10

**Authors:** Lida Wu, Quanyi Wang, Junzhong Gu, Huiyuan Zhang, Yuchun Gu

**Affiliations:** 1 Molecular Pharmacology Laboratory, Institute of Molecular Medicine, Peking University, Beijing 100871, China; 1301111601@pku.edu.cn (L.W.); gujunzhong@163.com (J.G.); zhanghuiyuan_2009@163.com (H.Z.); 2 Aston Medical School, Aston University, Birmingham B4 7ET, UK; 3 Department of Biopharmaceutics, School of Life Science and Technology, China Pharmaceutical University, Nanjing 210009, China; quanyiwang_@cpu.edu.cn

**Keywords:** actin filament dynamics, Kir2.1, filamin A, PIP_2_

## Abstract

Apart from its ion channel properties, the Kir2.1 channel has been found in tumors and cancer cells to facilitate cancer cell motility. It is assumed that Kir2.1 might be associated with cell actin filament dynamics. With the help of structured illumination microscopy (SIM), we show that Kir2.1 overexpression promotes actin filament dynamics, cell invasion, and adhesion. Mutated Kir2.1 channels, with impaired membrane expression, present much weaker actin regulatory effects, which indicates that precise Kir2.1 membrane localization is key to its actin filament remolding effect. It is found that Kir2.1 membrane expression and anchoring are associated with PIP_2_ affinity, and PIP_2_ depletion inhibits actin filament dynamics. We also report that membrane-expressed Kir2.1 regulates redistribution and phosphorylation of FLNA (filamin A), which may be the mechanism underlying Kir2.1 and actin filament dynamics. In conclusion, Kir2.1 membrane localization regulates cell actin filaments, and not the ion channel properties. These data indicate that Kir2.1 may have additional cellular functions distinct from the regulation of excitability, which provides new insight into the study of channel proteins.

## 1. Introduction

Precise temporal and spatial control of actin filament movements is key to multiple cellular processes, such as cell invasion [1], cell contraction [2], focal adhesion [3,4], and differentiation [5,6]. The actin filament network is not a fixed structure; two classes of actin filaments, characterized as dynamic and stable, have been found inside the cell. Dynamic actin filaments are capable of growing and shortening in dynamic instability [7]. Actin filaments have a different stability and contribute to different structures, which determine their functions [8]. To fulfill their functions, actin filaments continuously transition between stable and active forms [9]. As a result, it is vital to study actin filament transitions.

Andersen–Tawil syndrome (ATS, also known as long-QT7) is a channelopathy typically characterized by a triad of symptoms: cardiac arrhythmias, periodic paralysis, and dysmorphic features [10]. It is widely assumed that this disease is caused by the loss of function of Kir2.1 resulting from mutations [11,12]. However, several additional mutations in Kir2.1 have been identified in unrelated patients [10]. This suggests that Kir2.1 plays a role in additional developmental signaling in addition to its previously recognized function in controlling cell excitability. Aberrant expression levels of Kir2.1 channels have been detected in tumors and cancer cells, which facilitate cancer cell motility, invasion, and survival, the mechanism of which is associated with the cytoskeleton [13,14,15]. As a result, it is assumed that Kir2.1 has an actin regulatory effect.

Here, with the help of structured illumination microscopy (SIM), we reveal that Kir2.1 overexpression promotes actin filament dynamics, cell invasion, and adhesion. The actin-associated functions of the Kir2.1 channel depend critically on precise subcellular localization and the number of channel proteins on the cell surface membrane. In this process, PIP_2_ helps Kir2.1 membrane trafficking and its anchoring at the cell membrane, and membrane-expressed Kir2.1 promotes redistribution and phosphorylation of FLNA, which promotes orthogonal branching of actin filaments and links.

## 2. Results

### 2.1. Kir2.1 Regulates Actin Filament Morphology and Actin Dynamics

**Figure 1:**
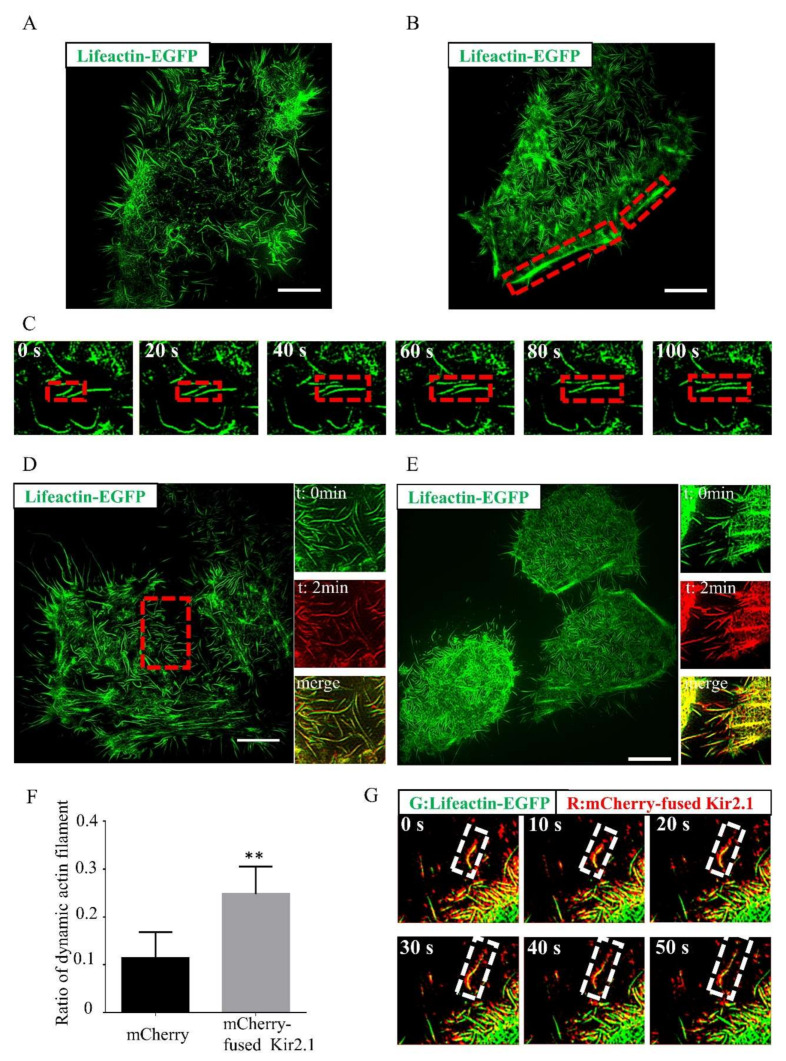
Kir2.1 regulates actin filament morphology and actin dynamics. (**A**,**B**) Actin filaments of Hela cells imaged by structured illumination microscopy (SIM). Hela cells were transfected with lifeact-EGFP and mCherry (**A**), lifeact-EGFP and mCherry-fused Kir2.1 (**B**), Scale bar: 5 μm. The red dotted frames show that actin filaments assembled in clusters. (**C**) Actin filaments in wild-type Hela cell imaged by SIM. Hela cells were transfected with lifeact-EGFP. Images in the red dotted frame show detailed growth of one actin filament. Most of the actin filaments are stable. (**D**,**E**) The dynamics of actin filaments in Hela cells imaged by SIM. Hela cells were transfected with lifeact-EGFP and mCherry (**D**), lifeact-EGFP and mCherry-fused Kir2.1 (**E**). Magnified images show detailed actin filaments. Actin filaments at different time frames were labeled with different pseudo colors (T = 0 min, green; T = 2 min, red); colocalized actin filaments represented the stable actin filaments in the merged image, while the non-overlapping actin filaments were dynamic ones. Scale bar: 5 μm. (**F**) Quantification of the dynamic actin filament ratio. The image of representative cells is shown in (D) and (E); *n* = 5 cells. Values are mean ± SEM. ** *p* < 0.01. (**G**) Movement of mCherry-fused Kir2.1 monitored by SIM. Hela cells were transfected with lifeact-EGFP and mCherry-fused Kir2.1. Magnified images show in the white dotted frame.

To analyze the effects of Kir2.1 on actin dynamics, Hela cells were transfected with a mCherry-fused Kir2.1 vector together with the actin label plasmid lifeact-EGFP. To retain its normal functional properties such as subunit assembly, trafficking, and rectification, Kir2.1 was fused to mCherry at the COOH-terminal end [16,17]. LifeAct is a peptide consisting of 17 amino acids comprising the actin-binding domain from yeast actin-binding protein 140 (ABP140) [18]. Its small size and absence from mammalian cells are ideal for binding F-actin with minimal disruption, allowing visualization of actin dynamics with minimal artifacts [19]. In order to achieve better temporal and spatial image resolution, SIM was employed in this study. With a spatial resolution of 88 nm, SIM offers twice the resolving power of diffraction-limited microscopy under relatively low doses of light compared with those required for other super-resolution modes, which makes it useful for hour-long time-lapse imaging of actin filaments in live cells [20]. mCherry empty plasmid transfected Hela cells presented long and tangle-some actin filaments (Figure 1A). Comparatively, the mCherry-fused Kir2.1-transfected cells presented more organized, straight actin filaments (Figure 1B), and some organized, straight actin filaments assembled in clusters along the cell edges (Figure 1B). Stable and dynamic actin filaments in Hela cells were also observed in this study. The dynamic actin filaments continually changed, while the stable actin filaments were relatively still (Figure 1C). We labeled the actin filaments at different time frames with different pseudo colors (T = 0 min, green; T = 2 min, red); colocalized actin filaments represented the stable actin filaments in the merged image, while the non-overlapping actin filaments represented dynamic ones. Hela cells were transfected with lifeact-EGFP and mCherry (Figure 1D), lifeact-EGFP and mCherry-fused Kir2.1 (Figure 1E). Most of the actin filaments were stable in Hela cells, and the dynamic ones were scarce (Figure 1D). Interestingly, the ratio of dynamic actin filaments increased significantly in the Kir2.1-overexpressed cells (Figure 1F).

The movement of Kir2.1 on the cell surface was also monitored continuously. Kir2.1 clusters did not move randomly as theoretically predicted. Many Kir2.1 clusters traveled along the dynamic actin filaments (Figure 1G), which indicated a specific connection between Kir2.1 and actin filament dynamics. It seemed that most Kir2.1 tended to locate along the adhesive cell edge or cluster in corners (Figure S1), where filopodia seemed most likely to emerge. In conclusion, Kir2.1 was associated with the processes of actin filament reorganization.

### 2.2. Kir2.1 Promotes Filopodial Extension, Cell Migration, and Cell Adhesion

**Figure 2:**
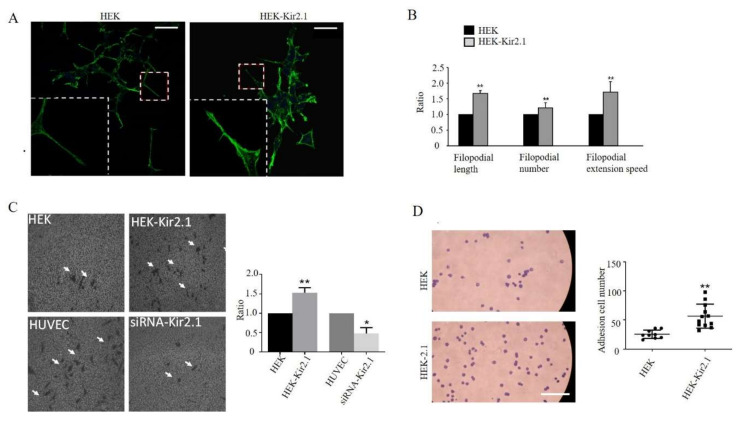
Kir2.1 promotes filopodial extension, cell migration, and cell adhesion. (**A**) F-actin was visualized by FITC-conjugated phalloidin staining in HEK and HEK-Kir2.1 cell lines. The white dotted frames show the magnified images. Scale bar: 100 μm. (**B**) Quantification of filopodial length, filopodial number, and filopodial extension speed. ** *p* < 0.01. (**C**) Representative fields of the Transwell invasion assay under a phase-contrast microscope. Cells were grown, transfected, and then subjected to the Transwell assay. The white arrows point to the migrated cells stained by methylrosanilnium chloride solution. Quantitative results of the relative ratio of migrated cells is shown on the right; ** *p* < 0.01, * *p* < 0.05. (**D**) Representative fields of the adhesion assay. Cells were stained by methylrosanilnium chloride solution and measured by counting after adhesion for 45 min. scare bar: 500 µm. Quantification of total adhesion cell number per random field is shown on the right; ** *p* < 0.01.

Cell invasion and adhesion are complex, multifaceted processes triggered by actin motor activity and are manifested by F-actin-based cytoskeletal core filopodial protrusion, adhesion, and so on [7,21]. Kir2.1 was therefore speculated to promote filopodial extension, cell invasion, and cell adhesion. To confirm this hypothesis, the HEK293A cell line was chosen in our experiments, as the HEK293A cell line presents relatively obvious, protrusive filopodia compared to Hela cells (Figure 2A). After 6 h growth on the same batch of glass plates, cells were fixated and stained with FITC-conjugated phalloidin; then, filopodial length, number, and filopodial extension speed were measured. Results showed that Kir2.1 promoted filopodial formation, extension, and increased filopodial extension speed (Figure 2A,B). Cell invasion capacity was also detected by Transwell assay. As Kir2.1 is highly expressed in HUVEC cells [22], and not expressed in HEK293A cells, we used these two cells lines for Transwell invasion assays. Overexpression of Kir2.1 in HEK293A promoted cell invasion, and Kir2.1 knockdown in human umbilical vein endothelial cells (HUVEC)s inhibited cell invasion (Figure 2C).

Similarly, using the cell adhesion assay, cells were stained with methylrosanilnium chloride solution and measured by counting after adhesion for 45min. Overexpression of Kir2.1 was found to promote cell adhesion (Figure 2D). To further explore the relevance between membrane Kir2.1 expression levels and cell adhesion capacity, Kir2.1 abundance on the HEK-Kir2.1 cell membrane was evaluated by the patch clamping technique, and the current density was used to represent the functional membrane channel expression level. It was found that cell adhesive capacity was positively related to Kir2.1 current density (Figure S2A,B), indicating that Kir2.1 promotes cell adhesion. In summary, Kir2.1 promotes cell filopodial extension, cell invasion, and cell adhesion.

### 2.3. Kir2.1 Membrane Expression and PIP_2_ Affinity Are Required to Facilitate Actin Filament Remolding

Kir2.1 function is critically dependent on the integrity of channel interactions with PIP_2_ [23,24]. Mutations in Kir2.1, which affect Kir-PIP_2_ binding, cause development of the genetic disease Andersen–Tawil syndrome (ATS) [10,25,26,27]. Del314/315 is a common mutation associated with ATS; deletion of amino acids at position 314 and 315 cause Kir2.1 Golgi apparatus retention, blocking Kir2.1 membrane expression [25]. Overexpression of Kir2.1-del314/315 had almost no effect on actin filament morphology and dynamic (Figure S3A,B), indicating that precise Kir2.1 membrane localization is key to its actin filament remolding effect.

**Figure 3:**
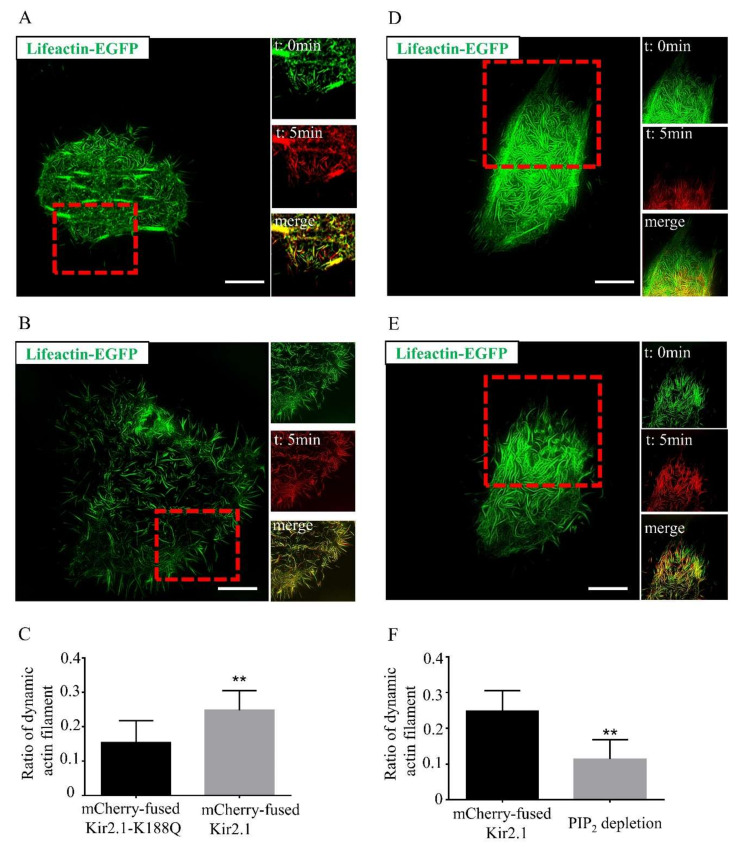
Mutations in Kir2.1 diminish the actin reorganization effect. (**A**) The dynamics of actin filaments in Kir2.1-overexpressed cells imaged by SIM. Hela cells were transfected with lifeact-EGFP and mCherry-fused Kir2.1. Scale bar: 5 μm. (**B**) The dynamics of actin filaments in the Kir2.1-K188Q-overexpressed cells imaged by SIM. Hela cells were transfected with lifeact-EGFP and mCherry-fused Kir2.1-K188Q. Scale bar: 5 μm. (**C**) Quantification of the dynamic actin filament ratio. Representative images were shown in (**A**) and (**B**). *n* = 3–4 cells. Values are mean ± SEM. ** *p* < 0.01. (**D**) The dynamics of actin filaments in Kir2.1-overexpressed cells before PIP_2_ depletion. Hela cells were transfected with lifeact-EGFP and mCherry-fused Kir2.1. Scale bar: 5 μm. (**E**) The dynamics of actin filaments in Kir2.1-overexpressed cell after PIP_2_ depletion. PLC agonist (10 µM m-3M3FBS) and PI3K inhibitor (100 nM wortmanin) were used as PIP_2_ depletion agents. Hela cells were transfected with lifeact-EGFP and mCherry-fused Kir2.1. Scale bar: 5 μm. (**F**) Quantification of the ratio of dynamic actin filaments in Kir2.1-overexpressed cells before and after PIP_2_ depletion. Representative images were shown in (**D**) and (**E**). *n* = 3 cells. Values are mean ± SEM. ** *p* < 0.01.

We also constructed another Kir2.1 expression plasmid bearing a K188Q mutation, which impairs membrane expression [28,29]. Hela cells were transfected with lifeact-EGFP and mCherry-fused Kir2.1 (Figure 3A), lifeact-EGFP and mCherry-fused Kir2.1-K188Q (Figure 3B). Kir2.1-K188Q mutation did not affect the actin filament morphology much and had a much weaker actin filament dynamic effect than wild-type Kir2.1 did (Figure 3C). As mutations in the Kir2.1 channel affect channel PIP_2_ affinity, cellular PIP_2_ was significantly depleted by the application of both PI_3_K inhibitor and PLC agonist in Kir2.1-overexpressed cells. It was then noticed that depletion of PIP_2_ not only dramatically changed the morphology of actin filaments (Figure 3D,E) but also blocked Kir2.1-associated actin filament reorganization (Figure 3F). As a result, both Kir2.1 membrane expression and PIP_2_ affinity are required to facilitate actin filament remolding, and PIP_2_ affinity may play a more crucial role in this process.

### 2.4. PIP_2_ Facilitates Kir2.1 Cell Surface Expression and Kir2.1 Movement at the Cell Membrane

**Figure 4:**
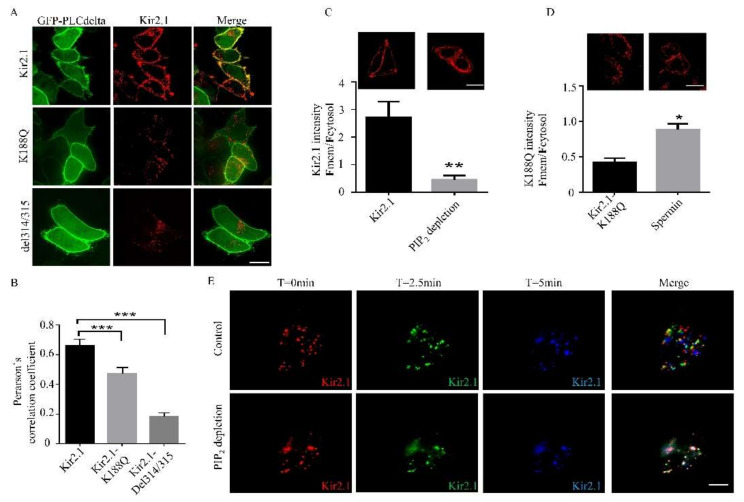
PIP_2_ facilitates Kir2.1 cell surface expression and Kir2.1 movement at the cell membrane. (**A**) Localization and distribution of PIP_2_ and Kir2.1 channels on the cell membrane. Hela cells were transfected with GFP-PLCdelta-PH and mCherry-fused Kir2.1 channels. PIP_2_ was traced by PLC delta fused with GFP. Scale bar: 10 μm. (**B**) Quantification of PIP_2_ -Kir channel co-localization by Pearson’s correlation coefficient. For *n* = 18–24 cells pooled across three independent experiments. *** *p* < 0.001. (**C**) Kir2.1 distribution in Hela cells before and after PIP_2_ depletion. Hela cells were transfected with mCherry-fused Kir2.1. Scale bar: 25μm. The quantification is shown below. *n* = 25–33 cells pooled across three independent experiments. ** *p* < 0.01. (**D**) Kir2.1-K188Q fused mCherry distribution in Hela cells cultured with or without spermin (100uM). Hela cells were transfected with mCherry-fused Kir2.1-K188Q. Spermin is a Kir2.1-PIP_2_ binding enhancer. Scale bar: 25 μm. The quantification is shown below. *n* = 19 cells pooled across three independent experiments. * *p* < 0.05. (**E**) Movement patterns of Kir2.1 before and after PIP_2_ depletion. Kir2.1 was labeled with the different pseudo colors at different time frames, and co-localization parts in the merged images represent the stillness of Kir2.1. Scale bar: 5 μm.

As Kir2.1-del314/315 and Kir2.1-K188Q mutations present impaired membrane expression and low PIP_2_ affinity, we assumed that PIP_2_ facilitated Kir2.1 cell surface expression. Hela cells were transfected with mCherry-fused Kir2.1 and PLCdelta-GFP. PLCdelta-GFP was used as a bio-tracker of PIP_2_. It was shown that both Kir2.1-del314/315 and Kir2.1-K188Q presented impaired surface expression, while wild-type Kir2.1 tended to colocalize with PIP_2_ at the cell membrane (Figure 4A,B). Besides, the depletion of PIP_2_ blocked Kir2.1 membrane expression (Figure 4C). Polyamines, such as spermin, strengthen the interaction between the Kir2.1 channel and PIP_2_ [30]. Spermin was shown to aid in Kir2.1-K188Q cell surface expression (Figure 4D). To test whether PIP_2_ facilitates the stability of Kir2.1 at the plasma membrane, the trafficking of Kir2.1 was also monitored. Hela cells were transfected with mCherry-fused Kir2.1, and Kir2.1 at different time frames was marked with different pseudo colors (T = 0 min, green; T = 2.5 min, red; T = 5 min, blue). Interestingly, the movement of Kir2.1 clusters on the cell membrane was significantly blocked when PIP_2_ was depleted (Figure 4E), and many Kir2.1 clusters gradually disappeared from the cell surface (Figure 4E). In summary, PIP_2_ facilitates Kir2.1 traffic towards, and stability at, the plasma membrane.

### 2.5. Kir2.1 Regulates Filamin A and p-Filamin A Redistribution

**Figure 5:**
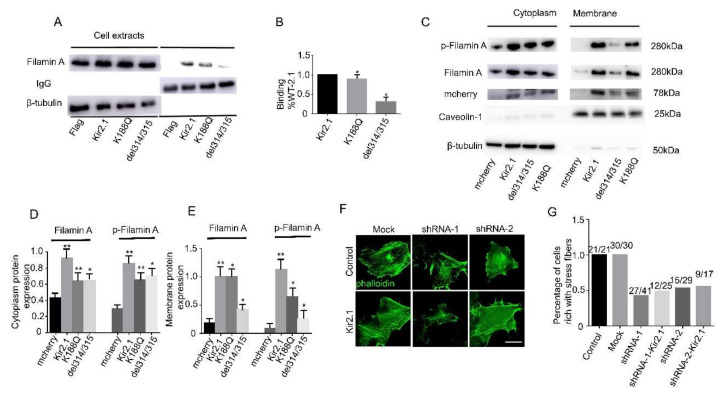
Kir2.1 regulates filamin A and p-filamin A redistribution. (**A**) Interaction between filamin A and Kir2.1 channels was detected by immunoblot (IB). (**B**) Quantification analysis of the binding capacity of Kir2.1 channels with filamin A. The K188Q and del314-15 mutations reduced the filamin A binding capacity. Values are mean ± S.D. * *p* < 0.05. (**C**) Hela cells were transfected with the mCherry empty backbone and mCherry-fused Kir2.1 channels. Plasma and membrane lysates of the mCherry empty backbone and mCherry-fused Kir2.1 channel expression cells were extracted and analyzed by immunoblotting with antibodies against filamin A, p-filamin A, mCherry, caveolin, and beta-tubulin. Beta-tubulin was used as a control for equal extraction of cell plasma proteins. Caveolin was used as a control for equal extraction of cell membrane proteins. (**D**,**E**) Quantification analysis of filamin A and p-filamin A expression and distribution in plasma and cell membranes. Values are mean ± S.D. ** *p* < 0.01, * *p* < 0.05. (**F**) F-actin was visualized by Alexa Fluor 488-phalloidin staining. Filamin A knockdown dramatically diminishes stress fiber formation. Scale bar: 10 µm. (**G**) Percentage of cells with rich stress fibers, which reflects the effects of filamin A knockdown on the organization of the actin filament system.

As Kir2.1 cell surface expression is a necessity to remold actin filaments, we speculated that Kir2.1 might act as a sponge to attract and relocate actin-binding proteins to specific locations on the cell membrane. Filamin-A (FLNA), an actin-binding protein that promotes orthogonal branching of actin filaments and links, interacts with Kir2.1 directly [31]. To detected the interaction between Kir2.1 and FLNA within cells, co-immunoprecipitation experiments were performed. Hela cells were transfected with Flag-tagged Kir2.1 (entire protein). Anti-Flag-conjugated beads directed against the Flag epitope on Kir2.1 were able to co-immunoprecipitate the FLNA (Figure 5A), suggesting that these two proteins form complexes within Hela cells. However, Kir2.1-del314/315 and Kir2.1-K188Q presented a weaker interaction (Figure 5A,B). To test whether Kir2.1 could regulate the localization of FLNA, the FLNA expression abundance on the cell surface and in plasma under Kir2.1 overexpression was examined by Western blot. β-tubulin and caveolin-1 were selected as plasma and membrane markers, respectively. It was found that Kir2.1 could increase FLNA cell surface and plasma expression, and the effect of Kir2.1-K188Q was stronger than Kir2.1-del314/315 (Figure 5C–E). Overexpression of Kir2.1 also increased FLNA phosphorylation and facilitated phosphorylated FLNA redistributed to the cell membrane (Figure 5C–E). Combining the co-immunoprecipitation experiments above, it was found that the stronger the Kir2.1-FLNA interaction, the higher the regulation effect of FLNA by Kir2.1. However, it was still unclear whether Kir2.1 could directly phosphorylate FLNA or bridge other proteins to the vicinity of FLNA. To further prove the actin remolding function of FLNA, Hela cells were transfected with FLNA shRNA, co-transfected with Kir2.1 and FLNA shRNA, and the stress fiber morphology change was analyzed using phalloidin staining. We used two sets of shRNAs to rollout the off-target effect. The results showed that stress fibers were significantly diminished in the FLNA-knockdown cells regardless of Kir2.1 expression (Figure 5F,G). Hence, to remold the actin filament network, Kir2.1 acts like a sponge attracting and relocating actin-binding proteins to specific locations on the cell membrane.

## 3. Discussion

Many ion channels interact with actin filaments or actin-associated proteins. However, the roles of these interactions remain incompletely understood. Most previous studies have concentrated on the regulatory effects of actin filaments on ion channels rather than the other way around. Many ion channels also regulate actin dynamics. As one example, Kv3.3 ion channels coordinate assembly of Arp2/3-dependent cortical actin networks by interacting with Hax-1 and Arp2/3 [32]. Our results also show the actin regulatory effect of Kir2.1, which further influences cell morphology and invasion ability, independent of ion channel electrical activity.

The normal function of ion channels depends critically on precise subcellular localization and the number of channel proteins on the cell surface membrane. Kir2.1 channels, bearing Andersen–Tawil syndrome mutations, are manifested as a disorder and characterized by ventricular arrhythmias, periodic paralysis, and skeletomuscular dysplasia [10]. Studies demonstrate that Kir2.1 channel properties play an essential role in the process [25,27]. Apart from its channel properties, our data present that aberrant Kir2.1 also has much weaker actin remolding effects, in comparison with wild-type Kir2.1, because of trafficking and localization problems. As defective potassium channel Kir2.1 trafficking underlies Andersen–Tawil syndrome [17], our results may provide a new mechanism to understand Andersen–Tawil syndrome, concerning aberrant myocyte contractions resulting from aberrant Kir2.1-associated actin remolding.

What process could be involved in Kir2.1 cellular trafficking and subcellular localization? Kir2.x channels associate with protein complexes that may be important to target and traffic channels to specific subcellular locations, as well as anchor and stabilize channels in the plasma membrane [33]. Kir2.1 channels incorporate into clathrin-coated vesicles at the trans-Golgi for export to the cell surface via its N- and C-terminal domains, which create an interaction site for the AP1 adaptin complex [25]. PIP_2_ has also been detected in clathrin-coated vesicles [34]. Proteins, such as PIK3C2A, regulating PIP_2_ metabolism are found to directly interact with clathrin and regulate clathrin-mediated membrane trafficking [35]. Consistently, our data indicate that PIP_2_ facilitates Kir2.1 surface expression. The depletion of PIP_2_ causes a decrease in the Kir2.1 current [30], the mechanism of which is obscure. Based on the observed phenomenon that depletion of PIP_2_ results in decreased Kir2.1 surface expression and membrane mobility, we suspect that PIP_2_ directs Kir2.1 anchoring at the cell membrane, and depletion of PIP2 causes Kir2.1 detaching from the cell membrane, which results in a decrease in Kir2.1 current.

Our results indicate that Kir2.1 cell surface expression is a necessity for actin filament dynamics. As was reported, Kir2.1 directly interacts with FLNA [31]. FLNA crosslinks actin filaments and also binds to actin-regulating RhoGTPases such as RhoA, Rac, and Cdc42 [36]. We speculated that interactions with Kir2.1 might regulate the location of FLNA on cell membranes and its vicinity to other proteins, which may influence its phosphorylation state. Our data show that overexpression of Kir2.1 redistributes both FLNA and phosphorylated FLNA between the cell surface and cytoplasm. However, the role of Kir2.1 in the FLNA phosphorylation process requires further investigation. A few G protein-coupled receptors can directly bind FLNA and promote FLNA phosphorylation [37]; small GTPase rho regulates Kir2.1 activity [38], and we believe that specific subcellular Kir2.1 may act as a bridge by accumulating proteins to some specific sites.

When it comes to ion channels, too much attention has been paid to their channel properties. The fact is often neglected that ion channels are also proteins, which will interact with and influence other molecules. Our work indicates that Kir2.1 membrane localization regulates cell actin filaments, but not ion channel properties. Kir2.1 may have additional cellular functions distinct from the regulation of excitability, which provides new insight into the study of channel proteins.

## 4. Materials and Methods

### 4.1. Cell Preparation

Hela cells were cultured in DMEM (Thermo Fisher Scientific, Waltham, MA, USA) with 10% FBS (Thermo Fisher Scientific). HeLa cells transfected with EGFP-Lifeact plasmids and KIir2.1-mCherry plasmids were cultured for 24 h. Then, cells were re-plated on coverslips for 5–8 h before imaging. Cells were directly imaged in PBS at room temperature.

HUVECs were grown in endothelial cell medium (Promocell) and maintained at 37 °C and 5% CO_2_. Cells were used before the fifth passage. HEK-293A was cultured (at 37 °C and 5% CO_2_) in DMEM medium (Thermo Fisher Scientific) with 10% FBS (Thermo Fisher Scientific).

### 4.2. Plasmids and cDNA Constructs

Kir2.1 constructs are derived from Kir2.1 cDNA (NM008425); Kir2.1 cDNA was subcloned into the pmCherry-C1 expression vector (Clontech, Santa Clara, CA, USA). The pmCherry-kir2.1-K188Q and pmCherry-kir2.1-del314/315 vectors were constructed with the Q5 Site-Directed Mutagenesis Kit (NEB, Ipswich, MA, USA). The primers are as follows:

mutK188Q-F, CAAAGCCAAAGCAGAGAAATGAGACTCTTGTCTTCAGTCAC;

mutK188Q-F, GTCTCATTTCTCTGCTTTGGCTTTGCCATCTTCGCCATGAC;

del314/315-F, CTCAATGCCGGAGTCTGGCCAATGAAATTCTCTGGGGTCACC;

del314/315-R, GAATTTCATTGGCCAGACTCCGGCATTGAGTTGTCATGGCAGTC.

Lifeact-mEGFP was a gift from Michael Davidson (Addgene plasmid # 54610).

GFP-C1-PLCdelta-PH was a gift from Tobias Meyer (Addgene plasmid # 21179).

### 4.3. The TIRF-SIM Setup

The schematic illustration of the system is based on a commercial inverted fluorescence microscope (IX83, Olympus) equipped with a TIRF objective (Apo N 100X/1.7 HI Oil, Olympus, Tokyo, Japan) and a multiband dichroic mirror (DM, ZT405/488/561/640-phase R; Chroma, Beijing, China). Laser light with wavelengths of 488 nm (Sapphire 488LP-200) and 561 nm (Sapphire 561LP-200, Coherent, Santa Clara, CA, USA) and acoustic optical tunable filters (AOTF, AA Opto-Electronic, Orsay, France) were used to combine, switch, and adjust the illumination power of the lasers. A collimating lens (focal length: 10 mm, Lightpath, Shanghai, China) was used to couple the lasers to a polarization-maintaining single-mode fiber (QPMJ-3AF3S, Oz Optics, Japan). The output lasers were then collimated by an objective lens (L1, CFI Plan Apochromat Lambda 2X NA 0.10, Nikon) and diffracted by the pure phase grating that consisted of a polarizing beam splitter (PBS), HWP, and the SLM. The diffraction beams were then focused on another achromatic lens (L2, AC508-250, Thorlabs) onto the intermediate pupil plane, where a carefully designed stop mask was placed to block the zero-order beam and other stray light and to permit passage of ±1 order beam pairs only. To maximally modulate the illumination pattern while eliminating the switching time between different excitation polarizations, a polarization rotator was built after the stop mask. Next, the light passed another lens (L3, AC254-125, Thorlabs, Shanghai, China) and a tube lens (L4, ITL200, Thorlabs) to focus on the back focal plane of the objective lens, which interfered at the image plane after passing the objective lens. Emitted fluorescence collected by the same objective passed through a dichroic mirror (DM), an emission filter, and another tube lens. Finally, the emitted fluorescence was split by an image splitter (W-VIEW GEMINI, Hamamatsu, Japan) before being captured by sCMOS (Flash 4.0 V2, Hamamatsu, Japan).

### 4.4. Confocal Imaging

Fluorescent images were acquired using a spinning disk confocal system (Revolution XD; Cold Spring Science Corporation, Cold Spring Harbor, NY, USA) with a CSU-X1 confocal head (Yokogawa, Japan) mounted on an inverted microscope (IX81ZDC2; Nikon, Minato, Japan) with Perfect Focus, using an EMCCD camera (iXon3 DU-897D-C00-#BV; Andor, Belfast, UK). Transillumination was provided by a halogen lamp and controlled by a SmartShutter (CSU-X1; Yokogawa). Confocal excitation was provided by a laser combiner with three laser lines at 488nm (OBIS; coherent), 561nm (OPSL; coherent), and 405nm (OPSL; coherent). The emission wavelength was controlled using a filter wheel (LB10W-2800; Sutter Instrument) outfitted with bandpass filters from Chroma Technology. Image acquisition and all other peripherals were controlled by MetaMorph.

### 4.5. Protein Preparation, Immunoprecipitation, and Immunoblots

Protein preparation—Total cellular protein was isolated with cell lysis buffer (CWBIO, Beijing China), and membrane and cytoplasmic protein fractions of cultured cells were obtained with a Mem-PER Plus Membrane Protein Extraction Kit (Thermo Fisher Scientific).

Immunoprecipitation—Flag fusion proteins were precipitated with anti-Flag-conjugated beads (Sigma, St. Louis, MO, USA). The proteins bound to beads were eluted with SDS sample buffer and analyzed by SDS-PAGE and quantitative Western blotting.

Immunoblots—Protein samples were loaded on an 8% or 10% polyacrylamide SDS gel and transferred to a polyvinylidene difluoride membrane (Millipore, MA, USA). The membranes were blocked in Tris-buffered saline (TBS; 20 mM Tris-Cl, pH 7.6, 137 mM NaCl) with 5% skim milk. Blots were incubated with the primary antibody diluted in TBS + 5% milk overnight at 4 C, washed three times in TBS + 0.5% Tween 20, and then incubated for 45 min in the peroxidase-conjugated secondary antibody. Blots were developed using the ECL detection kit.

The antibodies used in this study were as follows: anti-Filamin A (Abcam, Rabbit, 1:250,000), anti-Filamin A (phospho S2152) (Abcam, Rabbit, 1:10,000, Cambridge, UK); anti-Caveolin-1 (Proteintech, Rabbit, 1:5000, Wuhan, China); anti-mCherry (TDY bio, Mouse, 1:5000, Beijing, China); anti-Beta-tubulin (TDY bio, Mouse, 1:5000, Beijing, China).

### 4.6. Patch Clamping

Conventional whole-cell configurations of the patch-clamp technique were used in the electrophysiological study. Signals were amplified using an Axopatch 200B amplifier (Axon Instruments) and filtered at 1 kHz. Data acquisition and analysis were carried out using pClamp 9.0 (Axon Instruments, San Jose, CA, USA) software. Patch electrodes were pulled from a horizontal micropipette puller (P-1000, Sutter Instruments, Novato, CA, USA) and fire polished to a final tip resistance of 4–6 MΩ when filled with internal solutions. For whole-cell recording on HEK-293 cells, the pipette solution contained (in mM) 140 KCl, 2 MgCl_2_, 10 HEPES, 0.1 EGTA, 4 K2-ATP, and pH 7.3 (adjusted with KOH); the bath solution contained (in mM) 120 NaCl, 5 KCl, 1.5 CaCl_2_, 1 MgCl_2_, 10 HEPES, 10 glucose, and pH 7.4 (adjusted with NaOH). The data were acquired at 20 kHz and low-pass filtered at 5 kHz. During post-analysis, data were further filtered at 200 Hz.

### 4.7. Transwell Invasion Assay

Transwell invasion assays were performed according to the manufacturer’s instructions (Cell Biolabs Inc, Beijing, China). Briefly, the 24-well ECM-coated cell culture inserts were warmed at room temperature for 10 min. A cell suspension containing 1.0 × 10^6^ cells/mL was prepared in serum-free media. The cell suspension was placed in the upper chamber. Media (500 µL) containing 10% fetal bovine serum was added to the lower well of the plate and incubated for 2–24 h in a cell culture incubator. Finally, the cells were removed from the top of the membrane, and the migratory cells were stained and quantified.

### 4.8. Statistical Analysis

All statistical analyses and graphics were produced with GraphPad Prism 6 software (GraphPad Software, San Diego, CA, USA). Data sets were compared by unpaired two-tailed t-tests. Differences were considered statistically significant at *p* values below 0.05. * *p* < 0.05; ** *p* < 0.01; *** *p* < 0.001. All results are presented as mean ± SD or mean ± SEM.

## Supplementary Materials

The following figures are available online at https://www.mdpi.com/1422-0067/21/20/7479/s1. Figure S1 Movement of mCherry-fused Kir2.1 in Hela cells. (A) Movement of mCherry-fused Kir2.1 monitored by SIM. Hela cells were transfected with mCherry-fused Kir2.1. Kir2.1 was labeled with different pseudo colors at different time frames, co-localization parts (white) in the merged images represent the stillness of Kir2.1. Scale bar: 5 μm. Figure S2 Effect of Kir2.1 on cell adhesion. (A) Average I/V curve from whole-cell patch-clamp recording on HEK293A-Kir2.1 overexpression stable cell line. Cells were plated on glass plates for 15, 30, 45, and 60min before patch-clamp recording. (B) Histogram summarizing the current densities of HEK293A-Kir2.1 overexpression cells with different adhesion times. Figure S3 Mutations in Kir2.1 diminish the actin reorganization effect. (A) The dynamic of actin filament in Hela cells is imaged by SIM. Hela cells were transfected with lifeact-EGFP. Scale bar: 5 μm. (B) The dynamics of actin filament in Kir2.1-del314/315 overexpression cells imaged by SIM. Hela cells were transfected with lifeact-EGFP and mCherry-fused Kir2.1. Scale bar: 5 μm. (C) Quantification of the ratio of dynamic actin filaments. n=3 cells. Values are mean ± SEM. NS: no statistical significance.

## Abbreviations

ATS	Andersen–Tawil syndrome
F_cytosol_	fluorescence in the cytosol
FLNA	Filamin A
F_mem_	fluorescence in the membrane
GFP	green fluorescent protein
HEK293A	Human embryonic kidney 293 cells
FHUVEC	Human umbilical vein endothelial cells
Kir	inward rectifying potassium channel
PIP_2_	phosphatidylinositol 4,5-bisphosphate
PLC_2_	1-phosphatidylinositol 4,5-bisphosphate phosphodiesterase
SIM	structured illumination microscopy
