# Supplementary material for: Modulation of Actin Filament Dynamics by Inward Rectifying of Potassium Channel Kir2.1
Source: Int J Mol Sci. 2020 Oct 10;21(20):7479. doi: 10.3390/ijms21207479 (PMC7589188; doi:10.3390/ijms21207479)
Supplement: Supplementary file 1 [file ijms-21-07479-s001.pdf]

Modulation of actin filament dynamic by inward rectifying potassium channel Kir2.1

Lida Wu[1,3], Quanyi Wang[2], Junzhong Gu[1], Huiyuan Zhang[1], Yuchun Gu[1,3]*

1 Molecular Pharmacology Laboratory, Institute of Molecular Medicine, Peking University, Beijing100871, China.

2 Department of Biopharmaceutics, School of Life Science and Technology, China Pharmaceutical University, Nanjing 210009, China.

3 Aston Medical School, Aston University, Birmingham, B4 7ET, UK.

Address for correspondence:

Yuchun Gu, MD, PhD

Institute of Molecular Medicine (IMM), Peking University,

Room 216, Pacific Building, 52 Haidian Road，100871, Beijing, China

Email: wldpaper@pku.edu.cn

A

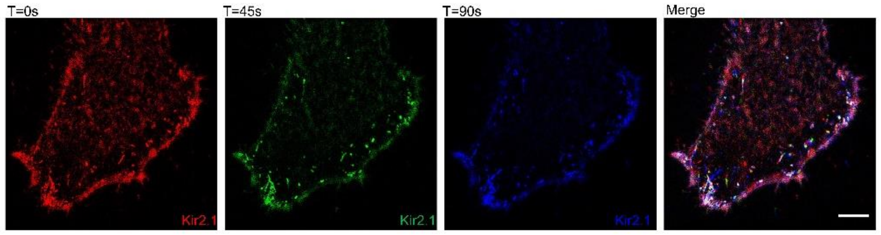

Supplementary Figure.1 Movement of mCherry-fused Kir2.1 in Hela cell. (A) Movement of mCherry-fused Kir2.1 monitored by SIM. Hela cells were transfected with mCherry-fused Kir2.1. Kir2.1 was labeled with different pseudo color at different time frames, co-localization parts (white) in the merged images represent the stillness of Kir2.1. Scale bar: 5 μm.

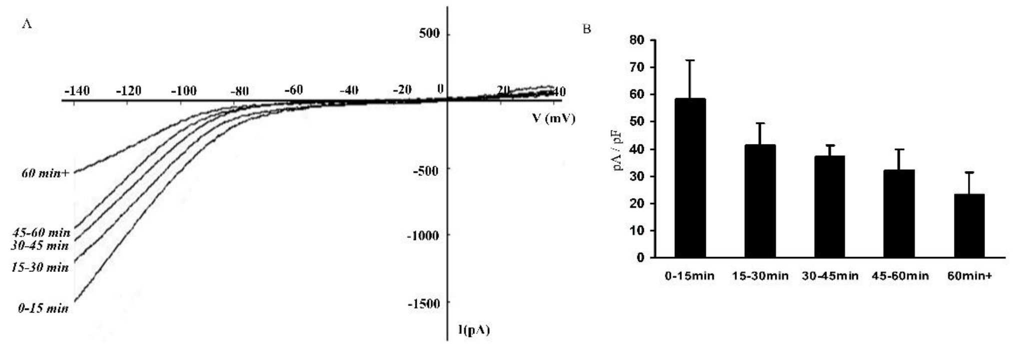

Supplementary Figure.2 Effect of Kir2.1 on cell adhesion. (A) Average I/V curve from whole-cell patch-clamp recording on HEK293A-Kir2.1 overexpression stable cell line. Cells were plated on glass plates for 15min, 30min, 45min, 60min before patch-clamp recording. (B) Histogram summarizing the current densities of HEK293A-Kir2.1 overexpression cells with different adhesion time.

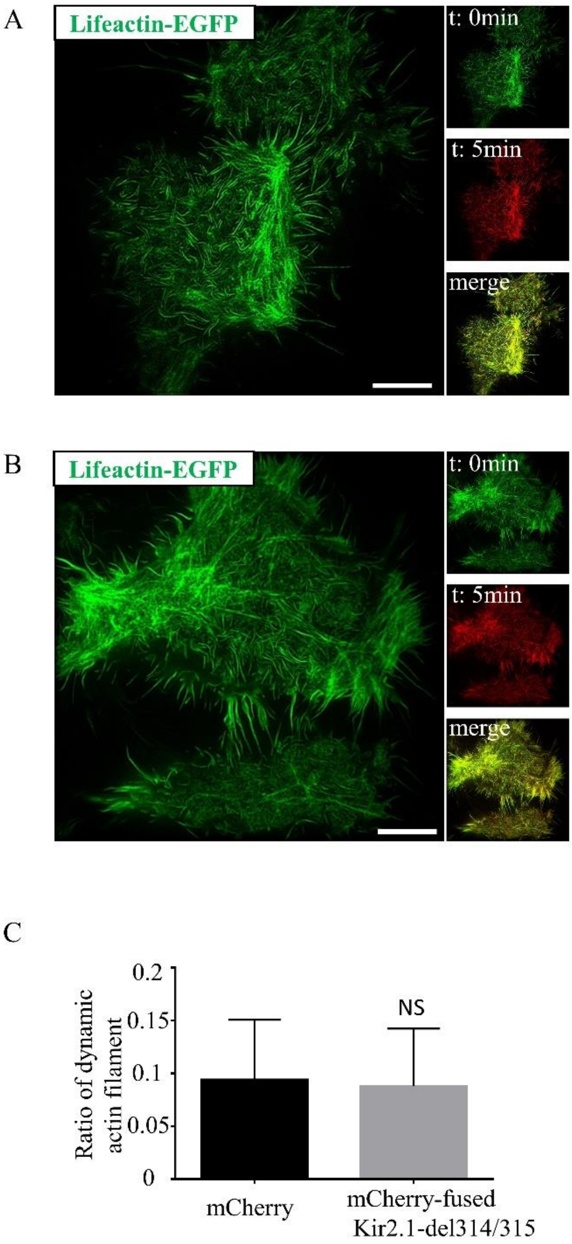

Supplementary Figure. 3 Mutations in Kir2.1 diminish actin reorganization effect. (A) The dynamic of actin filament in Hela cell imaged by SIM. Hela cells were transfected with lifeact-EGFP. Scale bar: 5 μm. (B) The dynamic of actin filament in Kir2.1-del314/315 overexpression cell imaged by SIM. Hela cells were transfected with lifeact-EGFP and mCherry-fused Kir2.1. Scale bar: 5 μm. (C) Quantification of the ratio of dynamic actin filaments. n=3 cells. Values are mean ± SEM. NS: no statistical significance.
